# Metformin-mediated effects on mesenchymal stem cells and mechanisms: proliferation, differentiation and aging

**DOI:** 10.3389/fphar.2024.1465697

**Published:** 2024-08-13

**Authors:** Xinjuan Liu, Zekun Li, Luyun Liu, Ping Zhang, Yue Wang, Gang Ding

**Affiliations:** School of Stomatology, Shandong Second Medical University, Weifang, Shandong, China

**Keywords:** mesenchymal stem cells, metformin, proliferation, differentiation, aging

## Abstract

Mesenchymal stem cells (MSCs) are a type of pluripotent adult stem cell with strong self-renewal and multi-differentiation abilities. Their excellent biological traits, minimal immunogenicity, and abundant availability have made them the perfect seed cells for treating a wide range of diseases. After more than 60 years of clinical practice, metformin is currently one of the most commonly used hypoglycaemic drugs for type 2 diabetes in clinical practice. In addition, metformin has shown great potential in the treatment of various systemic diseases except for type 2 diabetes in recent years, and the mechanisms are involved with antioxidant stress, anti-inflammatory, and induced autophagy, etc. This article reviews the effects and the underlying mechanisms of metformin on the biological properties, including proliferation, multi-differentiation, and aging, of MSCs *in vitro* and *in vivo* with the aim of providing theoretical support for in-depth scientific research and clinical applications in MSCs-mediated disease treatment.

## 1 Introduction

First isolated in mouse bone marrow by Friedenstein et al. in 1968, mesenchymal stem cells (MSCs) are nonhematopoietic stem cells derived from various postnatal tissues such as umbilical cord blood, fat, liver, skin, dental pulp, etc (Gronthos et al., 2000; Campagnoli et al., 2001; Keating, 2012), with the morphology similar to that of fibroblasts. MSCs possess profound multi-directional differentiation potentials and could differentiate into chondrocytes, osteoblasts, adipocytes under certain induced conditions (Longobardi et al., 2006). When flow cytometry is used to detect MSCs, it is found that the expression rates of CD105, CD90, and CD73 are above 95%, and the expression rates of CD45 and CD34 are below 5%. At the same time, if they meet the standard culture conditions for plastic adhesion and have the potential for multi-directional differentiation as mentioned above, the obtained sample can be considered as MSCs(Pittenger et al., 1999). MSCs are the most widely used postnatal tissue-specific stem cells used in clinical practice, and transplanting MSCs to replace damaged tissues can restore tissue structure and function (Keshavarz et al., 2023). In addition, MSCs were capable of secreting a large number of cytokines and growth factors, and playing multiple roles in immune regulation. Due to its characteristics of self-renewal, multi-directional differentiation potential, and low immunogenicity, MSCs have been considered as an excellent seed cells for regenerative medicine and tissue engineering and have been used in the research and treatment of various diseases (Sherman et al., 2019; Meng et al., 2020; Zheng et al., 2020; Hoang et al., 2022; Huang et al., 2023).

Metformin is orally administered, with a bio-availability of 50%–60%, and the plasma half-life has been calculated to be 1.5–4 h. It is absorbed through the intestine, enters the portal vein, and accumulates in the liver, while the remaining portion is excreted through urine and feces. In recent years, the efficacy and mechanism of metformin have been gradually elucidated, including but not limited to, improving blood glucose and insulin resistance, inhibiting the synthesis of insulin-like growth factor and aspartic acid, and affecting immune cells (Marcucci et al., 2020; LaMoia and Shulman, 2021; Veeramachaneni et al., 2021). As the most widely used anti-diabetic drug currently, metformin’s main role is to regulate blood glucose homeostasis and reduce insulin resistance by up-regulating adenosine 5′-monophosphate activated protein kinase (AMPK) to maintain cellular energy homeostasis and activate glucose uptake, thus being recommended as the first choice for treatment of type 2 diabetes patients (Wróbel et al., 2017). In addition to its anti-diabetes effect, more and more studies have also reported that metformin has unexpected efficacy in a variety of pathological and physiological processes, including but not limited to, alleviating oxidative stress and inflammatory reactions, treating several diseases, such as cancer, polycystic ovary syndrome, cardiovascular diseases, etc., with the potential to become a comprehensive therapeutic drug (Soldat-Stanković et al., 2021; Chaudhary and Kulkarni, 2024).

**FIGURE 1:**
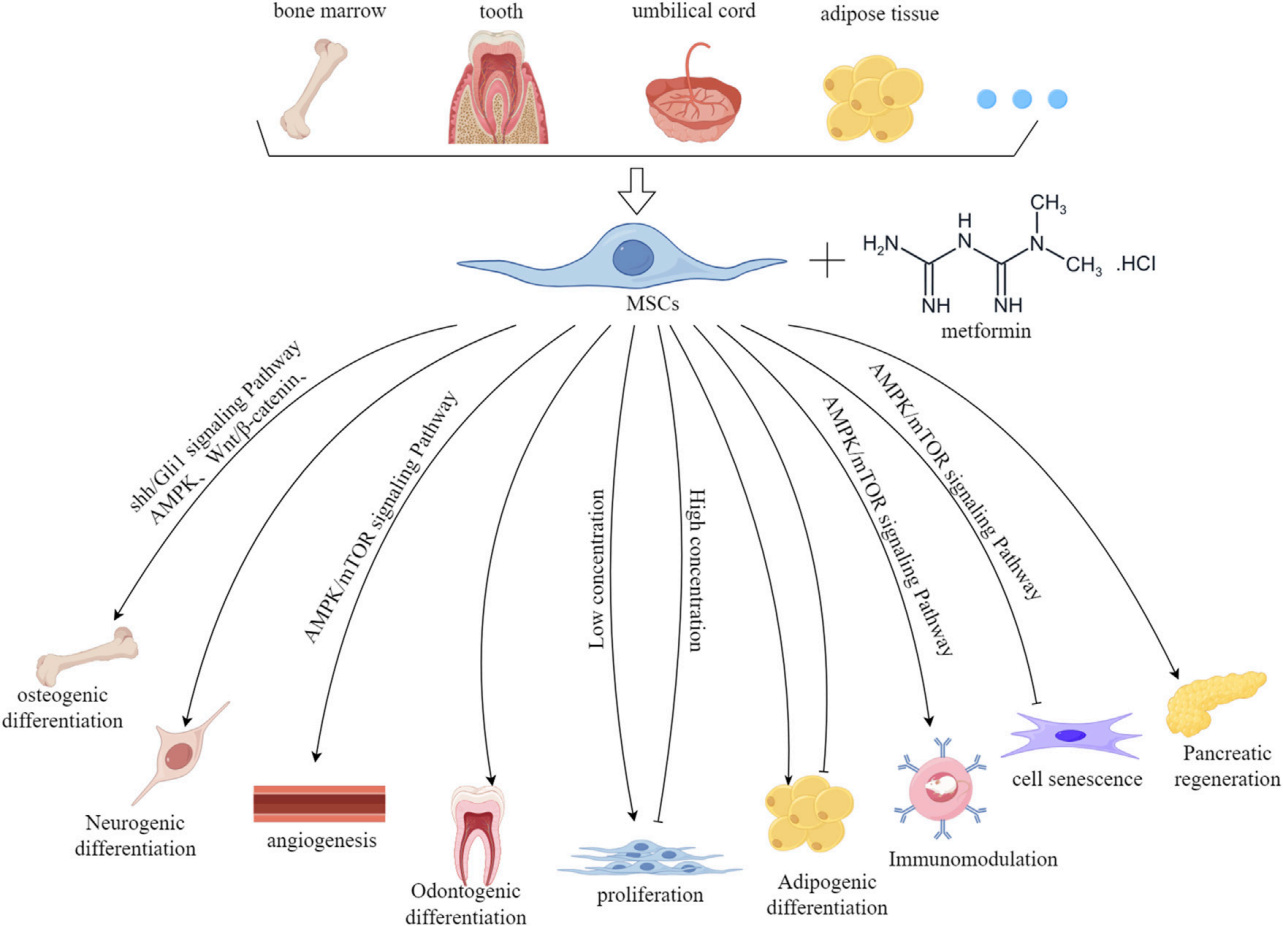
The effect and mechanism of metformin on the biological characteristics of MSCs derived from various tissue sources. This figure was generated by figdraw.

In recent years, a growing number of studies have shown that metformin has a significant effect on the biological properties of MSCs, including proliferation, multi-directional differentiation, and cell aging. Thus, it is necessary to explore the effects of metformin on MSCs in depth, therefore promoting their application in regenerative medicine. This article reviews the effects and possible mechanisms of metformin on the biological characteristics of MSCs (Figure 1).

## 2 The effect of metformin on the biological characteristics of MSCs *in vitro*

At present, *in vitro* studies on the biological functions of MSCs mainly focus on their proliferation, differentiation, and aging. In order to obtain sufficient numbers of MSCs for stem cell-based therapies, continuous cell culture and frequent passages are required (Liu, 2022). However, the number of cells obtained from conventional culture is insufficient for pre-clinical research and regenerative medicine applications, and repeated passages can also accelerate the aging of stem cells (Saei Arezoumand et al., 2016). It is therefore crucial to find effective methods to promote cell proliferation and differentiation and to inhibit aging.

### 2.1 The effect of metformin on the proliferation of MSCs

The effect of metformin on the proliferation of MSCs shows a double-edged sword phenomenon. Specifically, low concentrations of metformin promote the proliferation of MSCs, while high concentrations of metformin inhibit it (Table 1). The experimental results of Lei et al. indicated that 0–500 µM metformin has a promoting effect on the proliferation of human umbilical cord MSCs (UCMSCs), while high concentrations of metformin (1,000 µM) have inhibitory effects (Lei et al., 2021). However, in other experiments, when UCMSCs were treated with a higher concentration (0.1–50 mM) for 48 h, cell viability analysis showed that metformin caused a dose-dependent decrease in cell growth in all UCMSCs samples, reaching statistical significance from 3 mM and maximum efficacy at 50 mM (Bajetto et al., 2023). In another low concentration gradient experiment, metformin (10–200 µM) did not affect the viability and proliferation of MSCs from human exfoliated deciduous teeth (SHED) (Zhao et al., 2020), 10 µM metformin treatment had no significant effect on the proliferation of periodontal ligament stem cells (PDLSCs), while 50 μM and 100 µM metformin treatments significantly promoted the proliferation of PDLSCs on days 5 and 7 (Zhang et al., 2019). In addition, low concentrations of metformin (≤1,000 µM) was shown to have no significant influence on the proliferation of PDLSCs (Jia et al., 2020). This phenomenon may be due to different types of stem cells, different experimental conditions, and different observation times. In order to investigate the effect of processing time on MSCs, researchers have used different doses of metformin (0.5, 1, 10, 50, 100, 200, and 500 µM) to process bone marrow MSCs (BMSCs) for 14 days, and the results showed a dose-dependent decrease in cell survival rate, which may be due to the prolonged incubation time of metformin, leading to a decrease in the synthesis of factors such as insulin-like growth factor-2 (Montazersaheb et al., 2018). Under high sugar conditions, cell proliferation was significantly inhibited (Raghavan et al., 2020). However, it is worth noting that the addition of metformin reversed the inhibitory effect of high glucose levels on cell proliferation, mainly because metformin significantly cleared high glucose induced reactive oxygen species (ROS) (Dong et al., 2022) and regulated the ROS-AKT (protein kinase B)-mTOR (rapamycin target protein) axis inhibited by high glucose levels (Zhou et al., 2020). Previous studies have shown that biomaterials can be used to load metformin to further enhance its promoting effect on the proliferation of MSCs. Somayeh Ahmadi et al. encapsulated hyaluronic acid/gelatin with metformin loaded mesoporous silica nanoparticles, which resulted in a sustained release of metformin for 15 days. The authors co-cultured it with BMSCs and found that cell proliferation rate and metformin metabolic activity were improved through PicoGreen and 3 (4,5-dimethyl thiazol2-yl) 2, 5-diphenyl-tetrazolium bromide tests (Ahmadi et al., 2023).

**TABLE 1:** The effects of metformin on the proliferation of MSCs.

Cell sources	Metformin concentration	Action time	Effects	References
UCMSCs	0–500 µM	24 h	Promoted cell proliferation	Lei et al. (2021)
	>1000 µM		Inhibited cell proliferation	
UCMSCs	0.1–50 mM	48 h	Inhibited cell proliferation	Bajetto et al. (2023)
UCMSCs	10 µM	7 d	No significant influence	Al Jofi et al. (2018)
BMSCs	0.5–500 µM	14 d	Inhibited cell proliferation	Montazersaheb et al. (2018)
PDLSCs	≤1000 µM	5 d	No significant influence	Jia et al. (2020)
	2500 µM		Inhibited cell proliferation	
PDLSCs	10 µM	7 d	No significant influence	Zhang et al. (2019)
	50µM/100 µM	5d/7 d	Promoted cell proliferation	
SHEDs	10–200 µM	7 d	No significant influence	Zhao et al. (2020)
DPSCs	≤50 µM	7 d	No significant influence	Qin et al. (2018b)

Based on the studies conducted by the above researchers, it is possible to conclude that the proliferation of MSCs could be stimulated by a certain concentration of metformin. Further research is needed since the effects and mechanisms of metformin on cell proliferation are very complex, and different types of MSCs may respond differently to the drug. In addition, the response of these cells to metformin is closely tied to the duration of treatment.

### 2.2 The effect of metformin on multi-directional differentiation of MSCs

MSCs have the potential for multi-directional differentiation, and are capable of differentiating into chondrocytes, osteoblasts, adipocytes, neurons, and muscle cells under different induction conditions. Many researchers have demonstrated that metformin could promote or inhibit the multi-directional differentiation potential of MSCs to a certain extent and also explored possible mechanisms (Table 2).

**TABLE 2:** The effects of metformin on the differentiation of MSCs.

Cells	Biomaterial	*In vitro*	Dose of metformin	Results	*In vivo*	Dose of metformin	Animal models	Administration methods	Results	Mechanisms	References
rASCs	—	√	500 µM	Enhanced the odontogenic differentiation	√	250 mg/kg	Rat bilateral cranial defect mode	Drinking water	Promotes trabecular bone formation	Activating AMPK signaling pathway	Śmieszek et al. (2018)
iPSC-MSCs	—	√	10 µM	Enhanced the odontogenic differentiation	—	—	—	—	—	Activating LKB/AMPK signaling pathway	Wang et al. (2017)
rBMSCs	Metformin hydrochloride and citric acid	√	—	Enhanced the odontogenic differentiation	√	20 mg/kg	A periodontitis model of Wistar rats	The local injection	MCDs could more effectively enhance the periodontal bone regeneration than the raw material metformin	Activating ERK/AMPK signaling pathway	Ren et al. (2021)
N-BMSCs	—	√	50, 100 µM	Enhanced the odontogenic differentiation	√	50 mg/kg	Wistar rats femur defect model	Drinking water	Increase bone regeneration around implants	Activating AMPK/BMP/Smad signaling pathway	Sun et al. (2021)
DM-BMSCs	—		<200 µM	Enhanced the odontogenic differentiation (the maximum effective concentration is 125 µM)							
			≥200 µM	Inhibited osteogenic differentiation							
DPSCs	Demineralized dentin matrix	√	—	Increased mineralization and upregulated osteogenesis-related genes	—	—	—	—	—	Activating the AMPK pathway	Gao et al. (2020)
DPSCs	Calcium phosphate cement	√	50 μg/mL	Enhanced the odontogenic differentiation elevated expression of odontoblastic markers and strong mineral deposition	—	—	—	—	—	—	Qin et al. (2018b)
DPSCs	Freezedried bone allograft (FDBA)	√	100 µM	Enhanced the adhesion increase cell proliferation/viability Enhanced the osteogenic capability	—	—	—	—	—	—	Kouhestani et al. (2021)
DPSCs	Resin	√	20%	Enhanced odontoblastic differentiation Mineral synthesis	—	—	—	—	—	—	Wang et al. (2018)
PDLSCs	Polydopamine-templated hydroxyapatite (tHA)	√	100 µM	Promote osteogenic differentiation	—	—	—	—	—	Via the AMPK/mTOR signaling pathway by regulating autophagy	Yang et al. (2019)
			100 µM, 500 µM, 1 mM	Promote proliferation reduce cell apoptosis							
PDLSCs	Novel alginate-fibrin fibers	√	50 µM	Enhanced the odontogenic differentiation	—	—	—	—	—	Activating the Shh/Gli1 signaling pathway	Yin et al. (2023)
PDLSCs	—	√	10, 100, 500 µM	Promoted the osteogenic differentiation Inhibited the adipogenic differentiation	—	—	—	—	—	Activating of the Akt/Nrf2 signaling pathway	Jia et al. (2020)
PDLSCs	—	√	100 µM	Osteogenic differentiation under high glucose was decreased, and metformin addition enhanced it	—	—	—	—	—	Downregulation of NPR3 and inhibition of its downstream MAPK pathway	Zhang et al. (2022)
GMSCs	chitosan hydrogel	√	—	Powerful osteogenic, adipogenic and chondrogenic abilities in the induction medium supplemented with metformin	—	—	—	—	—	—	Cai et al. (2022)
SHEDs	—	√	100 µM	Promoted proliferation as well as osteogenic, adipogenic and chondrogenic differentiation Significantly improved the migration and angiogenesis of human umbilical vein endothelial cells	√	10 nM	Nude mice	The local injection	SHED pre-treated with metformin promotes angiogenesis	—	Deng et al. (2022)
BMSCs	—	√	100 µM	Enhanced the odontogenic differentiation	—	—	—	—	—	Inhibiting GSK3β activity Activating Wnt/β-catenin signaling	Ma et al. (2018)
UCMSCs	—	√	100 µM	Promotion of osteogenesis inhibition of adipogenesis Exhibited stronger angiogenesis	√	UCMSCs + metformin (100 µM)+ HUVEC	Male BALB/c nude mice	The local injection	Enhances the ability of angiogenesis	—	Lei et al. (2021)
UCMSCs	—	√	0.03–5 mM	A significant increase in lipid-rich vacuole formation No significant effect on osteogenesis	—	—	—	—	—	Activating the AMPK pathway	Bajetto et al. (2023)
ADMSCs	vitamin D3 (1nM and 2 nM)	√	25 mM	The combination of vitamin D3 and metformin accelerated the osteogenic differentiation of ADMSCs under high glucose concentrations	—	—	—	—	—	—	Ha et al. (2023)

#### 2.2.1 Osteogenic differentiation

Bone regeneration and repair are major challenges in clinical medicine, such as bone resorption caused by periodontitis and insufficient bone mass in implant repair in dentistry (Zhu et al., 2022). The use of metformin can reduce the risk of fractures in patients with type 2 diabetes, and is considered to play a direct role in promoting bone formation (Hidayat et al., 2019; Lee et al., 2019). Several studies investigated the effects of metformin on the osteogenic differentiation, detected the osteogenic markers, including alkaline phosphatase (ALP), osteocalcin and type I collagen after incubation of MSCs and metformin, and found the increased expression of these markers (Gao et al., 2008). However, the mechanism by which metformin promotes osteogenesis is not very clear. At present, many scientists believe that metformin mainly promotes osteogenic differentiation of MSCs through AMPK pathway.

Agnieszka et al. found that metformin significantly stimulated ALP activity and osteocalcin production, and promoted osteogenic differentiation of rat adipose tissue derived MSCs (ADMSCs) by activating AMPK signaling pathway. *In vivo*, using the rat bilateral skull defect model, the authors further observed that the amount of new bone and bone volume were significantly higher in the metformin group than in the control group (Śmieszek et al., 2018). Meanwhile, metformin at a concentration of 10 µM can induce osteogenic differentiation of pluripotent stem cell-derived MSCs (iPSC-MSCs), as shown by the significantly stimulated ALP activity, the increased expression of Runt-related transcription factor 2 (Runx2), and late-stage markers of osteoblasts, osterix. Functional organic cation transporter expressing iPSC-MSCs responded to metformin by inducing an osteogenic effect in part mediated by the LKB1/AMPK pathway (Wang et al., 2017), with the same pathway also found in another study on UCMSCs (Al Jofi et al., 2018). Nanomaterials have been widely used in various fields due to their outstanding advantages such as large surface area to volume ratio, high reactivity, and easy surface modification. Therefore, researchers have incorporated metformin into nanomaterials and investigated its effects on MSCs. Metformin carbon dots (MCDs) are prepared from metformin hydrochloride and urban acid via a hydraulic method. The experimental results showed that MCDs can activate the extracellular signal regulated kinase (ERK)/AMPK pathway, enhance the osteogenic potential of BMSCs, and their effect is superior to that of metformin. In a rat model of periodontitis, it was further observed that MCDs can effectively guide alveolar bone regeneration, while metformin cannot. Nevertheless, as a novel material, MCD has not been extensively studied in the field of MSCs, needing in-depth study to explore the effects and mechanisms (Ren et al., 2021).

Because metformin is a common drug used to treat type 2 diabetes, and type 2 diabetes patients are more likely to develop osteoporosis than non-diabetics, studies on how metformin promotes the osteogenic differentiation of MSCs are mostly conducted in diabetes models. When BMSCs from diabetic mice were treated with metformin, their osteogenic differentiation potential was enhanced, and when the metformin-treated cells were re-implanted into type 2 diabetic mice, their osteogenic effect was still good (Guo et al., 2023). Sun et al. treated BMSCs from alveolar bone of normal humans (N-BMSCs) and diabetes patients (DM-BMSCs) with different concentrations of metformin, and the results showed that the 50 and 100 µM groups significantly promoted osteogenesis of N-BMSCs, while in DM-BMSCs, metformin promoted ALP activity in a dose-dependent manner, with a maximum effective concentration of 125 µM. When the concentration exceeded 200 μM, metformin exhibited inhibitory effects on DM-BMSCs, owing to the lack of effective drug metabolism pathways in the cultured cell type and resulting in an inability to degrade metformin and subsequent drug accumulation and increased cytotoxicity, which is also the first evidence that high concentrations (>200 µM) of metformin can inhibit the osteogenic performance of DM-BMSCs. At the same time, *in vivo* experiments can observe that 50 and 100 µM metformin is able to promote bone integration indicators in rat bones and implants. In addition, metformin increased the expression of phosphorylated AMPK (p-AMPK) and Runx2, while compound C, a specific AMPK inhibitor, downregulated the expression of these proteins induced by metformin. Mechanism research suggested the AMPK/BMP/Smad pathway was responsible for metformin-promoted bone formation (Sun et al., 2021), which was also shown in Liang’s data (Liang et al., 2020).

Compared to other sources of MSCs, dental stem cells (DSCs) have the advantage of being easier to obtain and have a higher proliferation rate. Obtaining from discarded tissue also reduces ethical issues (Miura et al., 2003). Many researchers have also demonstrated that metformin promotes osteogenesis of DSCs (Qin et al., 2018a; Kuang et al., 2019; Zhang et al., 2019; Zhao et al., 2020). However, there is a phenomenon of rapid dilution when metformin is used alone, and more studies tend to combine metformin with other scaffold materials to develop a drug delivery system with controlled release of metformin, which has broader application prospects in clinical practice. Although as a nanobiomaterial that promotes bone tissue engineering and osteogenesis, high concentrations of polydopamine template hydroxyapatite (tHA) stimulate the production of ROS, leading to cell damage and apoptosis. Metformin can remove ROS and reduce its damage to cells. After the co-culture of PDLSCs + tHA + metformin, the expression of phosphorylated AMPK increased and the expression of phosphorylated mTOR decreased, indicating that the combination of tHA and metformin regulates autophagy through the AMPK/mTOR signaling pathway and thereby improving the survival ability of PDLSCs and further enhancing the osteogenic effect (Yang et al., 2019). When metformin was incorporated into a resin at 20% by mass as a model system, the gene expression levels of dentin sialophosphoprotein, dentin matrix hosphoprotein1, ALP, and Runx2 of dental pulp stem cells (DPSCs) were significantly increased compared to the group without metformin, which provides ideas for subsequent deep caries filling, pulp hole repair, and periodontal tissue regeneration (Wang et al., 2018). In addition, after loading metformin onto de-mineralized dentin matrix and co-culturing with DPSCs, an appropriate concentration of metformin could release regularly, and upregulation of osteogenic genes and increased mineralisation can be observed (Gao et al., 2020).

In addition to the AMPK pathway, other pathways also have been shown to promote osteogenesis in MSCs by metformin. The Wnt/β-catenin signaling pathway is involved in the regulation of various biological processes of life and has been proven to be a key signaling pathway in the osteogenic differentiation of BMSCs. Ma et al. showed that metformin (at a dose of 100 µM is the best) can increase the phosphorylation of GSK3β at serine 9 residue and promote the activation of β-catenin and the activity of TOPFlash, an effective tool to investigate the activation of Wnt signaling, therefore enhancing the metformin-mediated osteogenic differentiation of BMSCs (Ma et al., 2018). The same effects at the same concentration of metformin were also demonstrated in other studies (Jia et al., 2020; Zhao et al., 2020). In addition, inhibition of GSK-3β phosphorylation abolished metformin-induced osteogenic differentiation of BMSCs (Ma et al., 2018).

Metformin and PDLSCs were encapsulated in alginate fibrinogen solution to form alginate fibrinogen fibers, and 50 µM metformin promotes osteogenic differentiation of PDLSCs by up-regulating the Shh/Gli1 signaling pathway, while inhibiting the Shh/Gli1 signaling pathway significantly reduces the osteogenic differentiation ability of PDLSCs, which is the first evidence showing that the Shh/Gli1 signaling pathway is involved in metformin-enhanced osteogenic differentiation of PDLSCs. At the same time, it has been demonstrated that alginate fibrin embedded PDLSCs and metformin have great potential in the treatment of maxillofacial bone defects caused by trauma, tumors, etc (Yin et al., 2023).

The formation of bone tissue is influenced by various factors, among which the immune system is an important influencing part. Shen et al. co-cultured metformin-treated macrophages with UCMSCs and demonstrated that metformin plays a regulatory role in the conversion of M1 macrophages into M2 macrophages, and further enhanced M2 macrophages promote osteogenesis of UCMSCs by activating the PI3K/AKT/mTOR signaling pathway (Shen et al., 2022). In addition, the effect of metformin in promoting osteogenic differentiation of MSCs may also be related to treatment time. Experiments have shown that although 100 µM metformin can promote osteogenic differentiation of PDLSCs for 7 and 14 days, metformin significantly promoted the phosphorylation of AKT (p-AKT) at 10 min, and the degree of phosphorylation decreased with time up to 120 min. When the positive effect of metformin on p-AKT was inhibited, the expression of various osteogenic markers of PDLSCs was also reduced (Jia et al., 2020).

Although numerous studies have been conducted on the promotion of MSCs osteogenesis by metformin, and researchers have also proposed and tested its clinical application, it is currently limited to cell experiments and animal models. There is, therefore, a considerable way to go for further research.

#### 2.2.2 Lipogenic differentiation

It is generally accepted that osteogenesis and adipogenic differentiation are mutually exclusive processes. Following incubation of PDLSCs with metformin, the number of lipid droplets in the metformin group was found to be significantly reduced in comparison to the control group. The qRT-PCR results demonstrated that metformin treatment resulted in a reduction in the mRNA levels of the lipid production markers, including peroxisome proliferator-activated receptor γ (PPARγ) and the lipoprotein lipase, in a dose-dependent manner, which indicates that metformin inhibited the adipogenic differentiation of PDLSCs (Jia et al., 2020). Another *in vitro* experiment showed that 1 mM of metformin inhibited fat formation of BMSCs, which may be due to metformin-induced apoptosis of BMSCs and the filling of mouse tibial adipose tissue (Duan et al., 2022). However, Lei et al. have demonstrated that metformin at 3 and 5 mM enhances adipogenic differentiation in UCMSCs, leading to the upregulation of PPARγ mRNA and the downregulation of fatty acid binding protein 4 (Bajetto et al., 2023). Interestingly, similar results were also observed in an *in vivo* study, which showed that after 4 weeks of treatment, 200 mg/kg of metformin increased bone marrow adipose tissue in normal mice and ob/ob mice (a class T2DM model), and interfered with the proliferation of BMSCs and the expression of PPARγ mRNA in BMSCs.

#### 2.2.3 Neurogenic differentiation

Previous studies have shown that metformin activates an atypical protein kinase C- binding protein pathway, which promotes neurogenesis both in cells derived from the bodies of rodents and humans and in adult mouse brains in a CREB binding protein dependent manner (Wang et al., 2012). Although a number of experiments have demonstrated that MSCs exhibit a neural differentiation effect (Lai et al., 2022; Jaiswal and Dhayal, 2023; Romano et al., 2024), there is a paucity of studies investigating the effects of metformin on the neural differentiation of MSCs, and the majority of studies have focused on neural stem cells. Due to the origination from the cranial neural crest, DSCs are considered to have stronger neural differentiation potential than other MSCs, and are also commonly used in the research of neurodegeneration. A combination of chitosan hydrogel and metformin has been employed to enable metformin to function in a three-dimensional environment, significantly enhancing the expression of neural-related markers in human gingival MSCs, including nestin and β-microtubulin, and promoting the upregulation of nerve regeneration related proteins, including ATP5F1, ATP5J, NADH dehydrogenase Fe-S protein 3 and glutamate dehydrogenase 1, which proves the potential of human gingival MSCs-mediated treatment for neurological injury diseases (Cai et al., 2022).

#### 2.2.4 Odontogenic differentiation

Treatment of DPSCs with metformin has been demonstrated to increase the expression of dental cell markers, including dentin sialophosphoprotein and dentin matrix protein 1. Conversely, pre-treatment with compound C resulted in a significant reversal of odontogenic differentiation of DPSCs induced by metformin, indicating that an AMPK-dependent manner was involved in this process (Qin et al., 2018b). Furthermore, MCDs have been demonstrated to activate autophagy and enhance the odontogenic differentiation of DPSCs by up-regulating odontoblast gene marker and protein expression (Lu et al., 2022). When metformin is incorporated into dental resin, which is commonly employed in dental clinical practice, there is no discernible difference in the proliferation of DPSCs compared to resins without metformin. However, odontoblastic differentiation and mineral synthesis of DPSCs are greatly increased. Metformin resin exhibits no cytotoxicity and has the potential to stimulate pulp cells to synthesize new dentin and form dental bridges, demonstrating that the novel resin formulation comprising metformin and lining resin is anticipated to be employed in deep cavities and pulp capping, markedly enhancing the formation of tertiary dentin, facilitating pulp repair, and safeguarding the pulp (Wang et al., 2018). In addition, the combined application of calcium phosphate cement and DPSCs (Qin et al., 2018a), as well as that of novel alginate-fibrin fibers and PDLSCs (Yin et al., 2023), has also yielded an improvement in odontoblastic differentiation.

#### 2.2.5 Vascular differentiation

UCMSCs can facilitate the formation of blood vessels in the body by promoting the activity of human umbilical vein endothelial cells (HUVECs), and metformin can enhance this effect. In the experiment conducted by Lei et al., it was observed that UCMSCs treated with metformin exhibited a significantly enhanced angiogenic potential when co-cultured with HUVECs, which was evidenced by an increase in the expression of angiogenesis-related genes including stem cell factor, vascular endothelial growth factor receptor 2, and angiopoietin-2 in UCMSCs. Additionally, *in vivo* experiments using nude mice showed that metformin enhances the promotion of HUVECs angiogenesis by UCMSCs (Lei et al., 2021). BMSCs were incubated on fibrin scaffolds and implanted into the wound model of diabetes rats, and angiogenesis was promoted at the wound through the AKT/mTOR pathway. Moreover, the addition of metformin at an anti-diabetes dose resulted in a significant increase in the levels of p-AKT and p-mTOR in local diabetes wounds, therefore promoting the angiogenic potential of BMSCs (Du et al., 2023). Different concentrations of metformin (5, 10, 20 μM) were used to treat DPSCs, and a dose-dependent manner angiogenic potential of DPSCs was shown, thereby promoting the tissue regeneration mediated by DPSCs (Boreak et al., 2021). However, after long-term cultivation with metformin, the angiogenic potential of MSCs may decrease. Following 14 days of co-culture with BMSCs, the angiogenic potential of BMSCs is observed to decline, and suppression of BMSCs’ differentiating into the endothelial lineage by the overly active autophagy pathway was responsible for this phenomenon (Montazersaheb et al., 2018).

### 2.3 The effect of metformin on the aging of MSCs

**FIGURE 2:**
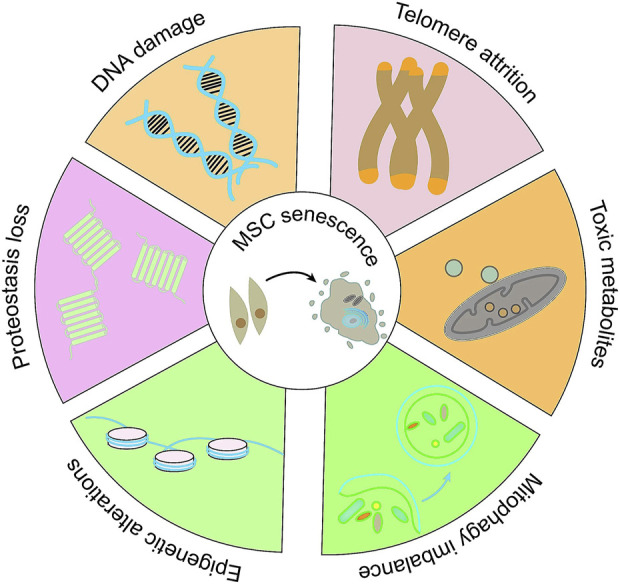
The molecular alterations in MSCs senescence. Adapted from (Zhao et al., 2024).

Cellular aging is a complex cellular state in which cells respond to various stressors, including changes in function and replication ability, ultimately leading to cessation of cell proliferation, lack of tissue recovery ability, and increased susceptibility to certain diseases (Figure 2) (Fasching, 2018; Zhao et al., 2024). Long term *in vitro* passage culture of MSCs could lead to replicative aging, weakened proliferation and differentiation ability, loss of surface antigen expression of MSCs, seriously affecting the function of MSCs(Wang et al., 2013). In addition, some exogenous stimuli can also induce premature aging of cells (Yu et al., 2021), resulting in decreased regenerative potential and affecting their application in regenerative medicine. It is of great importance for regenerative medicine to re-vitalize MSCs through various methods, in order that they could be re-used for possible treatments. Multiple experimental studies and epidemiological data have shown that metformin has great potential in the prevention and treatment of age-related diseases. Long term use can reduce many pathological risks associated with aging, including cardiovascular diseases, neurodegenerative diseases, and cancers (Porter et al., 2019; Schernthaner et al., 2022). Currently, research on the anti-aging effects of metformin is receiving increasing attention, and its impact on aging MSCs has also been extensively studied (Table 3).

**TABLE 3:** The effects of metformin on the aging of MSCs.

Cells	Model building	*In vitro*/*In vivo*	Dose of metformin	Treatment time	Conclusions	Mechanisms	References
BMSCs	20 g/L of D-gal following 24 h treatment	*in vitro*	10–500 µM 1, 2 mM	72 h	500 µM metformin exerted the best antiaging effect by targeting autophagy	—	Ye et al. (2023)
PDLSCs	exposed to 100 µM H_2_O_2_ for 2 h	*in vivo*	100 µM	24 h	Metformin could alleviate oxidative stress-induced senescence via stimulating autophagy	—	Kuang et al. (2019)
DPSCs	Aging donors	*in vitro*	100 µM	24 h	—	Upregulate CAB39 and activate the AMPK/mTOR signaling pathway by downregulating miR-34a-3p	Zhang et al. (2021)
rBMSCs	CKD mice	*in vitro*	10 µM	72 h	Metformin preconditioning may exhibit a therapeutic benefit to target accelerated senescence of CKD MSCs	—	Kim et al. (2021)
ADMSCs	Patients with CKD stage 5		Similar to that detected in the serum of patients with type 2 diabetes	—			
ADMSCs	Exposed to 200 µM H_2_O_2_ for 2 h	*in vitro*	100 µM	24 h	—	Conferred protection against H_2_O_2_-induced senescence by repressing the mTOR signaling pathway in an AMPK-mediated manner	Li et al. (2022)
		*in vivo*	—	—	Injection of metformin preconditioned ADSCs slowed osteoarthritis progression and reduced pain in mice		

#### 2.3.1 BMSCs

In a model of aging induced by D-galactose in BMSCs, metformin in a low concentration range (10–500 μM) has anti-aging effects on BMSCs, while the opposite is true in high concentrations (200 μM). The specific manifestation is that after treatment with metformin, the production of ROS, loss of mitochondrial membrane potential, and cell cycle arrest in aging cells are reversed, and further investigation revealed that activation of AMPK enhanced autophagy in aged BMSCs and inhibited the aging process (Ye et al., 2023). Compared with BMSCs extracted from normal rats, the ROS level of BMSCs extracted from diabetes rats was significantly higher, and the SA-β-gal staining results showed significant cellular aging. However, after the addition of 200 μM metformin, the intracellular ROS levels and cellular aging were significantly reduced, whereas addition of H_2_O_2_ inhibited the effect of metformin, indicating that metformin can inhibit cellular aging caused by oxidative stress (Dong et al., 2022). Furthermore, the combined application with biomaterials can achieve sustained release of metformin, thereby promoting the proliferation of BMSCs, and it has also been found that it could neutralize cell aging and attain adequate quantities of fresh BMSCs for tissue engineering applications (Ahmadi et al., 2023).

#### 2.3.2 DSCs

Following the treatment of PDLSCs with H_2_O_2_, a reduction in proliferation potential was observed, accompanied by an increase in lysosomal β-galactosidase activity, ROS accumulation and the expression of the senescence-associated secretory phenotype (SASP). Metformin pre-treatment has been demonstrated to stimulate autophagy of PDLSCs, partially reverse the harmful effects of H_2_O_2_ on PDLSCs. In addition, inhibiting autophagy with 3-methyladenosine has been shown to reverse the anti-aging effect of metformin (Kuang et al., 2019). When DPSCs were isolated from elderly donors, metformin was capable of down-regulating miR-34a-3p and up-regulating CAB39 via autophagy related AMPK/mTOR signaling pathway, thereby alleviating the aging of DPSCs (Zhang et al., 2021). However, another study showed that metformin could result in a significant increase in the number of senescent dental follicle cells. Concurrently, the ratio of NAD to NADH, an indicator of mitochondrial dysfunction and associated with the process of aging, was observed to decrease. These data demonstrated the complexity and uncertainty of metformin’s role in cellular aging (Morsczeck et al., 2023).

#### 2.3.3 Adipose derived MSCs

**FIGURE 3:**
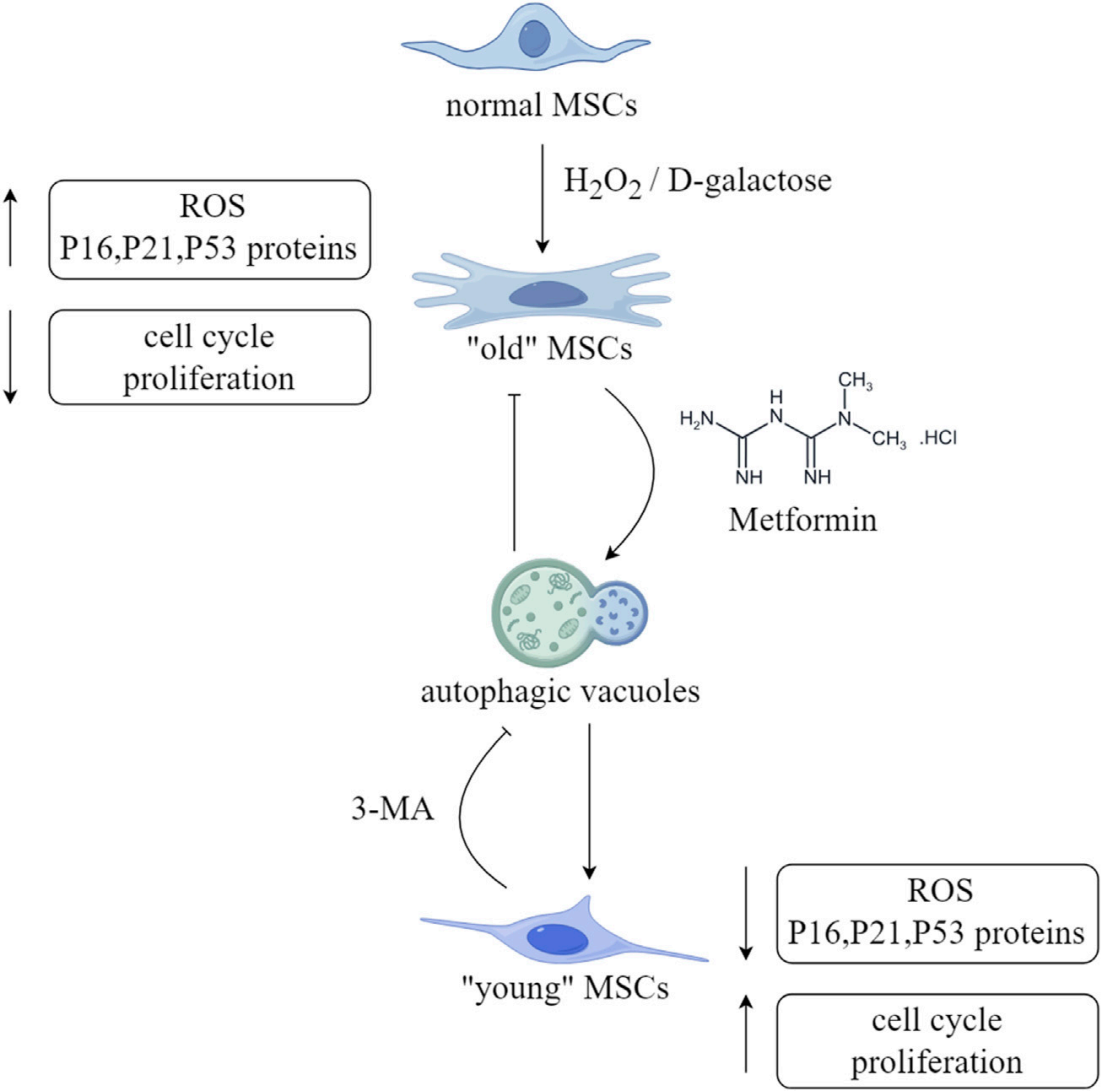
Schematic diagram of the anti-aging effect of metformin on MSCs. (ROS,reactive oxyge species). This figure was generated by figdraw.

After pre-treatment with metformin in H_2_O_2_ induced mouse ADMSCs aging model, the levels of ROS were significantly reduced, and the fluorescence intensity of DNA damage markers, including γH2A.X and Rad51, was also observed to be significantly decreased. In addition, the metformin-pretreated-ADMSCs group showed a significant increase in autophagic vacuoles, whereas the addition of 3-MA, an autophagy inhibitor, resulted in a decrease in the number of autophagic vacuoles (Figure 3). The same results were obtained by detecting protein expression levels. In addition, pre-treatment of ADMSCs with compound C resulted in increased levels of p21, p16, and p-mTOR, clearly displayed that metformin stimulates autophagy by activating the AMPK/mTOR pathway, alleviating H_2_O_2_ induced aging of ADMSCs (Li et al., 2022). Due to the high levels of chronic inflammation and oxidative stress in patients with chronic kidney disease (CKD), which can lead to the accumulation of aging cells, researchers isolated BMSCs from CKD mice and detected DNA damage and cell aging, and found that the expression of the LMNA gene and prelamin A increased, whereas metformin treatment for 72 h resulted in a reduction in their expression, shown by the upregulation of CDKN2A expression and SASP. Additionally, the same results were obtained by ADMSCs from CKD patients, proving that metformin treatment significantly weakened the effects of CKD on cell proliferation and aging. However, further research is required to elucidate the potential relationship and mechanism between aging, autophagy, ROS, and DNA damage (Kim et al., 2021).

## 3 The effect of metformin on the *in vivo* performance of MSCs

**FIGURE 4:**
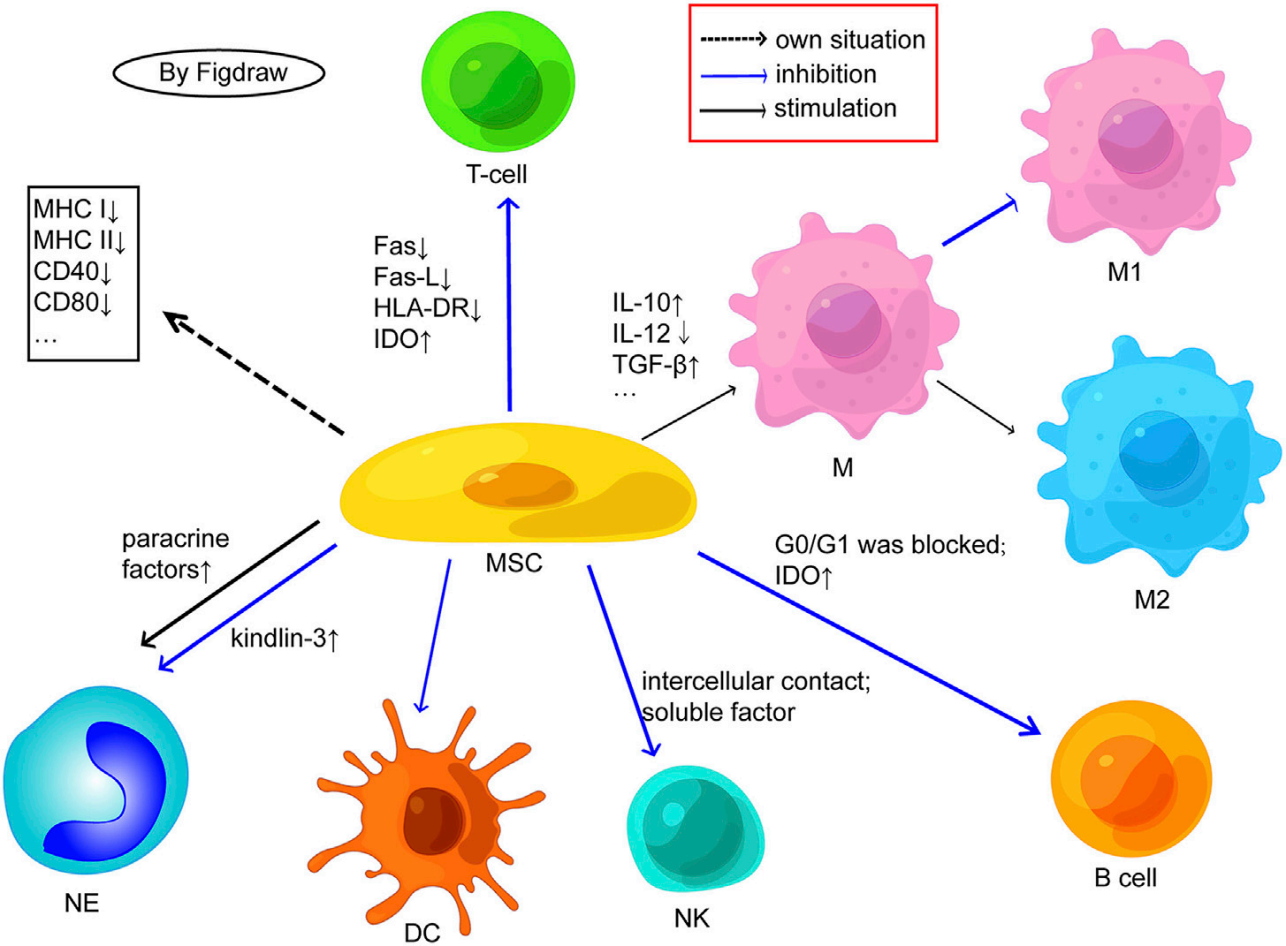
Possible cellular mechanisms related to the immunogenicity of MSCs. (M, macrophage; NK, natural killer cell; NE, neutrophil; DC, dendritic cells; Fas-L, fas ligand; IDO, indoleamine 2,3-dioxygenase; HLA, human leukocyte antigen; IL, interleukin; TGF, transforming growth factor). MSCs express low MHC/HLA class I and almost no MHC/HLA class II or costimulatory molecules. MSCs inhibit T cell proliferation by negatively regulating Fas ligand/Fas receptor, downregulating HLA-DR, and activating IDO; MSCs can also inhibit the differentiation of T cells into TH1 and TH17 and enhance the production of Tregs. MSCs facilitate the transition from M1 proinflammatory phenotype macrophages to anti-inflammatory phenotype M2 by promoting the secretion of anti-inflammatory factors and inhibiting the secretion of anti-inflammatory factors. MSCs may inhibit B cell proliferation by blocking G0/G1 phase and activating IDO. E, MSCs inhibit the proliferation of NK cells through intercellular contact and soluble factor mediation. MSCs have an inhibitory effect on the differentiation, maturation, and function of DCs. MSCs may maintain the viability and activity of neutrophils through paracrine factors and may also inhibit the release of NETs from neutrophils by upregulating kindlin-3 signalling. Adapted from (Chen et al., 2023).

The *in vivo* function of MSCs is mainly manifested in two principal aspects: immunity and regeneration. MSCs have a broad-spectrum immune regulatory ability, which can affect both acquired and innate immunity (Figure 4). It can promote inflammation when the immune system is under-activated, suppress inflammation when the immune system is over-activated to avoid self aggression (Aggarwal and Pittenger, 2005; Chen et al., 2023), and at the same time, MSCs secrete cytokines to promote or suppress immune responses and maintain immune balance according to the type or intensity of inflammation related signals (Jiang and Xu, 2019). In the context of an inflammatory microenvironment, MSCs can influence the proliferation, differentiation, and other functions of immune cells through cell-cell contact or the production of soluble factors, thereby facilitating the repair of damaged tissue, exerting anti-oxidant, immune regulatory, and anti-inflammatory effects, thus been widely used in the treatment of various autoimmune diseases (Lopez-Santalla et al., 2020; Gan et al., 2023). Meanwhile, MSCs can be safely harvested without major ethical issues, have low immunogenicity, and can diminish the risk of transplant rejection and complications, being of greater research value and regarded as an efficacious and reliable cell source for stem cell therapy (Wang et al., 2014). Metformin has been demonstrated to possess profound immune regulatory and anti-inflammatory effects *in vitro*, and has shown good therapeutic effects in the treatment of diseases such as cancer, inflammation, and endocrine disorders. In the microenvironment of the body, it can regulate mitochondrial function, autophagy, and immunity, significantly affecting the inflammatory state and health of the body (Cameron et al., 2016). Injury or aging may result in a decline in the body’s self-repair function (Notari et al., 2018). MSCs, when used alone or in conjunction with tissue engineering scaffolds, can facilitate tissue repair and play a pivotal role in regenerative medicine. Some progress has been made in regenerating human tissues using autologous MSCs(Lei et al., 2021; Zayed et al., 2021) (Table 4).

**TABLE 4:** The effects of metformin on the *in vivo* characteristics of MSCs.

Cells	*In vitro*	Dose and time of metformin	Gene expression	*In vivo*	Animals	Model induction	Concentration and time	Administration method	Results	Mechanisms	References
ADMSCs	√	5 mM 72 h	Enhanced IDO, IL-10 and TGF-β Inhibited CD4^−^ CD8^−^ T-cell expansion and Th17/Treg cell ratio	√	Female MRL/lpr mice	—	ADMSCs were pretreated with 5 mM metformin for 3 d	I.v.injection once weekly for 8 weeks	Significant disease activity improvement including inflammatory phenotype, glomerulonephritis, proteinuria and anti-dsDNA IgG antibody production	Metformin enhanced the immunomodulatory properties of Ad-MSCs through STAT1	Park et al. (2020)
UCMSCs	√	3 mM 72 h	Reduce the expression of IL-6, MCP-1, and COX-2	—	—		—	—	Reduce the production of inflammatory mediators, enhancing UCMSC immunoregolatory capacity on CD3^+^ and CD8^+^ T cells	—	Bajetto et al. (2023)
ADMSCs	√	1 mM 48 h	Increased IDO and IL-10 mRNA levels	√	Six-week-old male Wistar rats	Injected with 3 mg of monosodium iodoacetate to form osteoarthritis	—	ADMSCs or metformin stimulated ADMSCs were administered i.v. twice: at 0 and 4 days	Metformin pretreatment stimulated the immunoregulatory properties of ADMSCs and that this was associated with autophagy induction and improved mitochondrial function	—	Park et al. (2019)
BMSCs	√	—	IL-6 and IL-1 expression downregulated and Tgfβ and IL-10 expression upregulated in a dose-dependent manner	√	Male C57BL/6 mice	3% DSS in drinking water was provided *ad libitum* for 7 days	—	The tail vein injection	Metformin could improve the impaired therapeutic efficacy of OE-Nap1l2 BMSCs in the treatment of colitis and experimental autoimmune encephalomyelitis in mice	Metformin treatment could rescue the immunomodulation capacities of BMSCs by activating the AMPK signalling pathway	Liu et al. (2024)
BFPMSCs	—	—	—	√	Male and female Wistar rats with weights 200–250 g and ages 3–4 months	Injecting streptozotocin intravenously with a dose of 45 mg/kg of body weight	BFPMSCs were pre-treated with 1 mM metformin for 24 h	Injected intramuscularly into rats at 0.5 millioncells/animal	Metformin preconditioned BFPMSCs can regenerate pancreatic cells	—	Kamble et al. (2023)
SHED	√	100 μM 24 h	SHED pre-treated with metformin improves migration and angiogenesis of HUVECs *in vitro*	√	Nude mice	—	—	Injected into the lower dorsal region of the nude mice	SHED pre-treated with metformin exhibits a strong capacity for promoting angiogenesis	—	Deng et al. (2022)
BMSCs	—	—	—	√	Male adult Wistar rats (150–170 g)	Injected with streptozotocin (52.5 mg/kg) to Induce diabetes	300 mg/kg/d after diabetes con-firmation at day 3 and continued till the end of the study	Oral gavage	Metformin impairs homing and engraftment of transplanted BMSCs in the heart	Metformin treatment led to activation of AMPK that further was responsible for poor homing and survival of transplanted BMSCs in the heart	Ammar et al. (2021)
BMSCs	√	50 and 100 μM 14 d	The angiogenic potential of MSCs was shown to decrease after prolonged incubation with metformin	—	—	—	—	—	—	—	Montazersaheb et al. (2018)

### 3.1 Metformin enhances the immunomodulatory properties of MSCs

Metformin has been demonstrated to regulate metabolism, with the potential to treat autoimmune diseases such as systemic lupus erythematosus (Titov et al., 2019), chronic colitis (Takahara et al., 2022), Sjogren’s syndrome (Kim et al., 2019), and others. Metformin pre-treatment resulted in an increase in indoleamine 2, 3-dioxygenase and IL-10 mRNA levels in the culture supernatant of ADMSCs, which suggested that it could upregulate the immune regulatory properties of ADMSCs. In addition, metformin exposure to ADMSCs can lead to the appearance of autophagic vacuoles, and an increase in mitochondrial cristae and density, indicating that metformin pre-treatment stimulated the immune regulatory properties of ADMSCs, which is related to autophagy induction and improved mitochondrial function. Concomitantly, the expression of p-AMPK and p-STAT1 is upregulated, while that of p-STAT3 and p-mTOR is inhibited, demonstrating that metformin promotes the immunomodulatory capacity of ADMSCs by enhancing the expression of STAT1, which depends on the AMPK/mTOR pathway. Subsequently, researchers used lupus animal models to study the *in vivo* effects of metformin on ADMSCs, and the results demonstrated that metformin significantly mitigated the clinical features of lupus, nephritis, and immune cell dysfunction in mice by regulating CD90.2^+^CD4^−^CD8^−^ double-negative T cells, CD4^+^IL-17^+^ T cells, and CD4^+^CD25^+^Foxp3^+^ regulatory T cells, indicating that ADMSCs treated with metformin possess a comprehensive immune regulatory capacity, which may have potential in the clinical application of MSCs for lupus cell therapy (Park et al., 2020). Furthermore, metformin has been shown to significantly enhance the immune regulatory ability of UCMSCs on CD3^+^ and CD8^+^ T cells. Nevertheless, the conditioned medium of UCMSCs treated with metformin is ineffective against CD4^+^ cells, as these cells require different inflammatory cytokines to provide a stimulating response, suggesting that the effect of metformin on the immunoregulatory capabilities of MSCs is closely related to the pro-inflammatory factors (Bajetto et al., 2023).

Nucleosome assembly protein 1-like 2(Nap1l2) significantly impairs the migration and immune regulatory ability of BMSCs. Transplanting Nap1l2 over-expressing BMSCs into mice reduces the therapeutic effect on autoimmune diseases, including inflammatory bowel disease and experimental allergic encephalomyelitis. However, metformin has been shown to increase the AMP/ATP ratio, activate the AMPK signaling pathway, affect the metabolism of OE-Nap1l2 BMSCs, and enhance their immune regulatory ability (Liu et al., 2024).

### 3.2 Metformin enhances the regenerative properties of MSCs

MSCs are considered a potential therapeutic agent in regenerative medicine, and the formation of regenerative tissues is dependent upon the presence of blood vessels (Hou et al., 2015). As previously stated, UCMSCs can facilitate HUVECs angiogenesis *in vivo*, and metformin can augment this effect (Lei et al., 2021). Prajakta Kamble et al. conducted an experiment to test the efficacy of metformin-pretreated buccal fat pad-MSCs (BFPMSCs) in reversing hyperglycaemia. In the animal experiment with diabetic rats as the model, it was found that the blood sugar of the rats in the stem cell group, following pre-treatment with metformin, was reduced in comparison to the rats in other groups. Additionally, the pancreatic and serum insulin levels were also normal. The histological results demonstrated that pancreatic cells increased, and the pancreatic regeneration ability was reversed, indicating that the BFPMSCs pre-treated with metformin could regenerate pancreatic cells (Kamble et al., 2023). In comparison to the control group, HUVECs co-cultured with SHED that had been pre-treated with metformin demonstrated the capacity to form more capillary-like structures, showing a higher anti-CD31-positive capillary density (Deng et al., 2022). Nevertheless, it should be noted that not all metformin pre-treated MSCs demonstrate beneficial effects in disease models. Transplantation of BMSCs can prevent cardiac fibrosis and promote angiogenesis in diabetic rats. However, adding metformin could downregulate the cardiac protection mediated by BMSCs. Interestingly, both BMSCs and metformin alone can improve heart function, while the combination of metformin and BMSCs does not have a synergistic effect. As for the underlying mechanisms, metformin treatment was shown to impair the homing of transplanted BMSCs in the heart and lead to a decrease in the survival rate of transplanted cells, which is caused by AMPK activation (Ammar et al., 2021).

## 4 Existing problems and future direction

MSCs are a promising tool in the field of regenerative medicine due to their ability to proliferate, differentiate into multiple cell types, regulate the immune system, and exhibit other beneficial characteristics. Their versatility and therapeutic potential have led to their extensive use in various disease models. Nevertheless, in the treatment of certain pre-clinical models, the requisite characteristics are not consistently expressed, necessitating the optimization of MSCs applications to ensure optimal outcomes. Metformin has good bio-safety and relatively low cost, and has been widely used in clinical diagnosis and treatment for many years. Recent studies have demonstrated that its effects extend beyond the regulation of blood sugar. A review of existing clinical and experimental studies indicates that metformin has excellent bone promoting effects, inhibits cell aging, and has anti-tumor effects. Nevertheless, the study of metformin based on its non-antidiabetic functions is still in its infancy, which suggests that metformin still has considerable scope for further investigation in this field. The current studies are mainly limited to pre-clinical cell and animal research models. Following the demonstration of numerous positive effects *in vitro* and in animal studies, it is recommended that human clinical trials be conducted in order to evaluate the extent to which metformin has beneficial effects on human participants. Meanwhile, compared to single drug therapy, the combination of drugs using stem cells may have better therapeutic effects. However, it is worth noting that the optimal concentration of metformin varies greatly among different cell types. Exploring the appropriate concentration of metformin for different cells is of great significance for its application in tissue engineering. Metformin exerts its effects on MSCs through a multitude of molecular mechanisms and signaling pathways, which may vary depending on the source of the stem cells. Furthermore, the sustained effect of metformin in applications is also a significant issue that requires further investigation, and the combined use of scaffold materials may be a good choice. Anyway, the positive effects of metformin on tissue regeneration of MSCs still require more *in vivo* experiments to verify, which is a key area of future research.

## 5 Conclusion

Metformin, a commonly used drug, has many potential applications and considerable benefits on the biological characteristics of MSCs, providing broader insights for MSCs-mediated immunomodulation and tissue regeneration. However, there is still a long way to go before the application in clinics.
